# An Orangutan Hangs Up a Tool for Future Use

**DOI:** 10.1038/s41598-018-31331-7

**Published:** 2018-08-27

**Authors:** Nicholas J. Mulcahy

**Affiliations:** 1 School of Psychology, McElwain Building, University of Queensland, St. Lucia, Brisbane Queensland Australia; 2 Present Address: World Ape Fund International House, 124 Cromwell Road, London, SW7 4ET United Kingdom

## Abstract

Observational reports suggest that great apes perform tool-orientated actions in preparation for a tool’s future use. Some of these observations suggest remarkable planning skills because the target for the tool’s intended use was not present during the tool-orientated actions. Although these observational reports are intriguing, such planning ability has yet to be studied experimentally. To address this issue, I conducted two experiments that were inspired by an orangutan’s innovative behaviour during a novel enrichment task: the orangutan spontaneously secured a tool he was using to rake-in rewards by hanging it up when it was not in use but would be required a short time later. Experiment 1 showed that securing the tool predominately occurred when the orangutan could anticipate the tool’s future use, but rarely occurred when he could anticipate no further use for it. Experiment 2 indicated that the tool’s atypical size and/or weight were possible factors that prompted the orangutan to hang up the tool. Overall, the findings suggest that an orangutan not only innovated a novel way of securing a tool, but did so in anticipation of its future use.

## Introduction

Experimental research findings suggest that great apes have the ability of foresight by saving tools for future use^1,2^. In these studies, great apes were able to select, transport and return with a tool that they could predict would be needed hours later for obtaining food rewards. It is, however, contentious if these findings represent examples of foresight. One alternative explanation is that associative learning can better explain the findings^3,4^. That is, the great apes did not save the appropriate tool in anticipation of its planned future use, but they saved it because they had simply formed a strong association between the tool and gaining rewards during pre-experimental training procedures. In support of this criticism is that none of the subjects tested showed any evidence of specific tool-orientated behaviours that indicated they were anticipating the tool’s future use. Subjects, for instance, did not secure the tool against loss or hide it from their competitors, factors that resulted in subjects often losing the tool^4^. However, observational reports suggest that apes do perform tool-oriented actions in anticipation of a tool’s future use. Examples include wild orangutans (*Pongo abelii*) making appropriate short tools prior to entering a cemengang tree to extract seeds from its fruits^5^, wild chimpanzees (*Pan troglodytes verus*) sharpening sticks prior to using them to spear prey^6^ and a captive chimpanzee (*Pan troglodytes*) periodically attacking zoo visitors with stones that he had apparently cached in anticipation of the visitors’ arrival^7,8^. Observational reports are, however, difficult to assess without additional experimental evidence. What is therefore needed are experimental studies that test if apes can demonstrate tool-orientated behaviours that occur before the object for the tool’s intended use is visible. Here, I report a study that addressed this factor. The study was conducted with a zoo-housed adult Sumatran orangutan (*Pongo abelii*) called Riau. During a novel enrichment tool-use activity, that involved raking-in rewards with a long and heavy tool, Riau spontaneously hung up the tool by wedging it into narrow gaps of his enclosure’s mesh fence. The possible advantages of securing the tool were that Riau did not have to keep holding the heavy tool or he did not have to keep climbing down to pick up the tool if he dropped it to the floor after raking-in the rewards. Riau secured the tool during the periods of the enrichment task in which the tool was not immediately required to rake-in rewards but would be required a short time after its last use. I conducted an experiment to investigate if Riau might have been securing the tool in anticipation of its future use. This involved showing Riau rewards that were inside an opaque container and during regular intervals placing two of the rewards out of his reach that he could rake-in with the tool. Each time Riau had obtained the rewards, the container/rewards were hidden from his view for a short period of time. When the last rewards were placed out of his reach, Riau was shown that the container was empty before it was occluded from his view. I predicted that if Riau could anticipate the tool’s future use he would only secure the tool during the waiting period when he knew that there were rewards left inside the food container because an empty container would indicate there were no further rewards pending.

I conducted a second experiment that was similar to experiment 1 and included an additional condition that involved a much smaller and lighter tool. This investigated if the large size and/or heavy weight of the tool used in experiment 1 were factors that might have prompted Riau to secure the tool during the waiting periods. If these factors prompted tool securing, I expected Riau would be less likely to secure the smaller and lighter tool than the larger one.

## Results

### Experiment 1

As can be seen in Table 1, Riau secured the large tool in Experiment 1 in 98% (59/60) of Hang-up Tool trials in which he could predict its future use for raking-in forthcoming rewards. By contrast, there was a low frequency of observed large tool securing (8%, 1/12) in the Discard Tool trials in which Riau could predict no future use for the tool as no further rewards were pending. The difference was significant (Exact *X*^2^ Test, *X*^2^ = 58.32, p < 0.0001). The trial outcomes were significantly different between the first trial responses where 11/12 (92%) of first Hang-up Tool trials resulted in large tool-securing, whereas only 1/12 (8%) of Discard Tool trials resulted in large tool-securing (McNemar Exact Test, p = 0.002, N = 12).

**Table 1:** Tool type and number of occasions it was secured after its last use where Riau could be expected to predict the tool’s further use (Hang-up Tool trials) or no further use for the tool (Discard Tool trials).

	Exp 1	Exp 2
Large tool in first 5 Hang-up Tool trials	59/60 (98%)	30/30 (100%)
Large tool in last Discard Tool trials	(1/12) 8%	0/6 0%
Small tool in first 5 Hang-up Tool trials	Not tested	0/30 (0%)
Small tool in last Discard Tool trials	Not tested	0/6 (0%)

### Experiment 2

In Experiment 2 a similar contrast was seen within the frequencies of large tool trials. Riau secured the large tool in 100% (30/30) of the Hang-up Tool trials but did not secure the large tool in any of the 6 Discard Tool trials (Exact *X*^2^ Test, *X*^2^ = 36.00, p < 0.0001). By contrast, Riau never secured the small tool in the Hang-up Tool trials (0%, 0/30) or in the 6 Discard Tool trials (no difference, p < 0.99). There was, likewise, a clear consistency in the difference of first trial responses in the Large Tool condition where all 6 first trials in the Hang-up Tool trials resulted in tool-securing whereas none of the 6 Discard Tool trials resulted in tool securing, (Exact McNemar Test, p = 0.031, N = 6).

#### Qualitative Analysis

LEAVE/STAY behaviour: during the 1-minute period after Riau had raked-in the rewards, he stayed at the testing table in 96.7% of trials in experiment 1 (n = 60 Large Tool) and 100% of the trials in experiment 2 (n = 30 Large Tool, n = 30 Small Tool). By contrast, in the Discard Tool trials, Riau left the testing table 100% of the time when tested with the Large Tool in experiment 1 and 2 (n = 12 and n = 6, respectively), and 83% of the time in experiment 2 when tested with the small tool (n = 6).

WAITING behaviour: when Riau stayed at the table during the 1-minute waiting period, he never made any food begging gestures towards the experimenter in experiment 1 or 2. His general behaviour in these trials was to sit quietly facing the experimenter whilst gazing in the direction of the experimenter and around the testing area, for example to his left or right or down towards the table.

TOOL HOLD/DROP behaviour: in the 1-minute waiting periods between the Hang-up trials and when Riau did not hang up the tool, he kept hold of the tool 100% of the time in the Large Tool condition (n = 1) and 100% of the time in the Small Tool condition (n = 30). After Riau had raked in the rewards of the Discard Trials, he let go of the large tool in 100% of the trials (n = 18) in experiment 1 and 2 whereas he kept hold of the small tool 100% of the time (n = 6) in experiment 2.

## Discussion

The findings of this study suggest that an orangutan spontaneously developed a novel way of securing a tool in anticipation of its future use. Riau repeatedly hung up a large and heavy tool when it was possible for him to predict that it would be required a short time after its last use but rarely hung it up when he could predict no further use for it. Several of Riau’s behaviours throughout testing support that he was anticipating the future use of the tool. For instance, Riau rarely left the testing table when he could anticipate forthcoming trials in which he could rake-in rewards with the tool he had either kept hold of or hung up. Also, when Riau stayed at the testing table, during the waiting periods, he always sat quietly, facing in the direction of the experimenter and never made any food-begging gestures. If Riau had made such gestures, it may have suggested that he was staying at the testing table in an attempt to elicit rewards from the experimenter rather than anticipating that the experimenter would place further rewards on the table. Finally, Riau did not wait at the test table in 94% of the final trials in which he could anticipate that no more rewards would be forthcoming. However, in these trials he behaved differently in regards to holding or discarding the tool depending on the type of tool he was using. Before leaving the testing table, Riau always discarded the large tool at the testing area by dropping it to the floor or sometimes leaning it against the fence next to the table. In contrast, he always took the small tool when leaving the testing area. It could be argued that Riau had a strong preference per se for the small tool and this is why he never hung up the small tool in experiment 2. It is therefore possible that Riau was not contemplating the small tool’s future use. However, it was noted that when Riau took the small tool, he discarded it very soon afterwards. It is possible, then, that the small size and light weight of the small tool meant that there was little cost in leaving with the tool whereas the cost of doing so with the large tool may have been greater because of its bigger size and weight. This greater cost in weight and size may also explain why Riau spontaneously developed his securing strategy. Securing the large and heavy tool would overcome the costs of holding it during the waiting periods. The results of experiment 2 support this hypothesis. Riau always secured the larger tool when it was required for forthcoming trials, but he did not secure the small and lighter tool on any occasion.

Previous studies showing that apes can save tools over long time delays for future use have failed to demonstrate that any subject performed tool-directed behaviours that indicated they were anticipating the future use of the tools they were required to save^1,2^. It would therefore be interesting to conduct such planning studies using tools that might prompt subjects to perform tool-directed behaviours. A large cumbersome tool, as used in the current study, may prompt subjects to secure it rather than to carry it around. Another possibility is to use a fragile tool that may prompt subjects to take particular care of it during the waiting period. More research is therefore needed that allows subjects to demonstrate behaviours that indicate that they are anticipating a tool’s future use. The current study is a step in the right direction and demonstrates an orangutan may anticipate a tool’s use for the very near future. But more research is needed to test if such tool safekeeping extends to the more distant future.

In conclusion, the finding that Riau hung up a tool during the waiting periods in which the tool was not required suggests he did so in anticipation for the tool’s future use. Further research is needed to investigate if this type of tool-orientated behaviour extends to other ape species, and if they can perform tool-orientated behaviours for the distant future.

## Materials and Methods

### Subject

Riau, a 20-year-old zoo-born and mother-reared Sumatran orangutan (*pongo abelii*) housed at Singapore Zoo, participated in this study. Riau was tested in his daytime enclosure (238 m^2^). The enclosure contained planted vegetation, climbing frames and rope swings. Riau received daily enrichment of sunflower seeds scattered among leaves that were on the enclosure’s floor, and twice per day he was provided with branches with edible bark and leaves. Riau could also make tools from the branches to rake-in fruit that was placed twice per day inside Plexiglass enrichment boxes that had small holes in the lids. Riau had many years’ experience of using and making tools to rake rewards out of such enrichment boxes, however this always involved a keeper filling the enrichment boxes with rewards (before Riau entered the enclosure) and simply allowing Riau to retrieve the rewards ad libitum. Riau had previously participated in a tool-use study in which he was required to discriminate between a functional and non-functional tool, such as a broken versus an unbroken one^9^. None of the five orangutan keepers had previously witnessed Riau, or any other orangutan housed at Singapore Zoo, securing tools. Two of these keepers have been caring for Riau since his birth.

It is worth noting that in the wild not all orangutan species use sticks as tools to the same degree and no orangutan species has ever been observed raking in out-of-reach food items. Riau developed the ability to use sticks to rake-in food rewards in captivity and therefore it could be argued that the raking task is not suitable to test the cognitive abilities of orangutans because it lacks ecologically validity. This might be the case for Bornean orangutans as they have rarely been observed in the wild using sticks as tools. For example, in an 8-year observational study there was only one documentation of a wild Bornean orangutan using a stick as a tool. This involved a male orangutan breaking off a branch and using it to scratch himself^10^.

However, for Sumatran orang-utans, like Riau, raking-in out-of-reach rewards may be more ecologically valid as they appear to be more skilful than Bornean orangutans at using sticks as tools in the wild. This is especially the case when it comes to using tools for acquiring food, such as using small sticks to remove seeds from fruit and poking sticks into tree holes to obtain insects or their products^11^. Also, a male Sumatran orangutan was observed breaking off a branch and using it to hook onto another tree branch to aid his locomotion between the trees^12^. Therefore, although raking out-of-reach rewards has not been reported in orangutans living in the wild, the above tool-use behaviours of the Sumatran orangutans are similar to using sticks to rake-in rewards. Moreover, Sumatran orangutans rescued from the wild and living in a rehabilitation centre easily developed the skill to rake-in rewards when tested with a raking task^13^. Therefore although raking-in rewards is a skill that appears to develop only in captivity, it involves several tool actions that are ecologically valid for Sumatran orangutans.

The research was approved by the University of Queensland’s Native/Exotic Wildlife and Marine Animals Committee (reference PSY/313/09) and was conducted in accordance with all animal welfare laws of Australia and Singapore. Riau had access to drinking water throughout the study via an automated drinking nozzle within his outside enclosure and there was no disruption to his normal feeding times that occurred three times per day: morning, afternoon and early evening. Riau’s daily diet consisted of fruit, vegetables, seeds, fruit juice, boiled rice and a boiled egg. At all times, Riau was able to choose to stop participating in the enrichment task and experiments.

#### Enrichment Task

A wooden testing table (100 × 50 cm) was attached to the outside of the subject’s enclosure’s mesh fence at a height of 130 cm above the enclosure’s floor. On the inside of the mesh fence, a log was secured horizontally 118 cm above the floor allowing Riau to sit or stand directly in front of the table during enrichment tasks or cognitive studies. This also allowed zoo visitors to have a clear view of him participating in such activities.

As part of a new enrichment program, the experimenter placed rewards inside a metal ring (0.71 Kg, 8 cm diameter and 7 cm in length) that was positioned on the table 20 cm out of Riau’s reach. To gain access to the rewards, Riau had to make a tool to rake-in the ring and then remove it with his fingers allowing him to pick up the rewards. On the first occasion Riau encountered the task, he manufactured a tool (stripped the branch of leaves and small stem shoots) from a shrub growing within his enclosure and near to the table. The tool, however, was not strong enough to move the ring and after several unsuccessful attempts with the unsuitable tool, Riau ventured farther inside his enclosure (~10 meters) to access a larger shrub from which he obtained a stronger and larger tool (0.32 Kg, 1.42 m long, 2.1 cm thick at one end tapering to a final thickness of 0.4 cm). Riau used this stronger tool to successfully rake-in the metal ring and obtain the rewards. Over the next 40 min the experimenter periodically (~2 min) moved the metal ring back to its original position and replenished it with rewards (that were stored out of the subject’s view) at which point Riau invariably raked-in the baited ring with the same functional tool he had made to solve the task.

**Figure 1:**
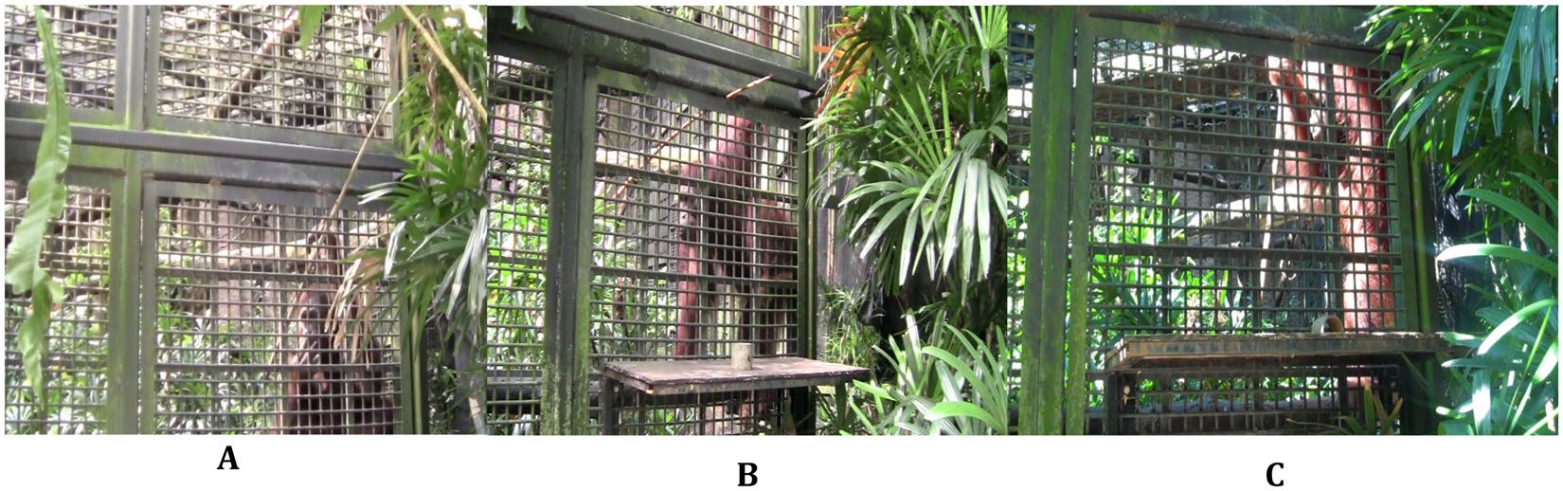
Riau securing the tool for future use and discarding it when it is not required. (**A**) Riau is balancing the tool inside the cage lattice in the early stages of the enrichment sessions. (**B**) In the later stages of the enrichment sessions, Riau wedges the tool inside the narrow gap, which secures the tool quickly and robustly. Hanging up the tool this way was the final method that Riau developed and one he persisted with during test trials in which he could predict its further use. (**C**) Riau is dropping the tool to the floor in the experimental trials when he can anticipate no further use for it.

During the time that Riau waited for the experimenter to reposition and replenish the ring, he initially held the tool or dropped it over 1 m to the floor picking it up once he had the opportunity to rake-in the ring/rewards. However, midway through the enrichment session, Riau adopted a different strategy during the waiting periods. He inserted the tool through one square (4 cm in length and width) of the cage lattice and manipulated the position of the tool until it balanced inside. Riau repeated this behaviour several more times during the waiting periods of the enrichment trials. Balancing the tool inside the lattice was difficult because it had to be specifically manipulated. This involved inserting the tool through the lattice and then letting go so that one end of the tool tipped down as the other end rose. If Riau correctly manipulated the tool it would catch within the lattice and become lightly wedged. On one occasion, Riau was attempting to balance the tool inside the lattice but then removed the tool and inserted it into a part of the lattice that was narrower (Fig. 1A). This made it easier to wedge the tool because there was less space for the tool to move around before catching within the lattice. Riau secured the tool in the narrower lattice two more times during the enrichment trials before developing another method of wedging the tool into an even more narrow gap (0.7 cm) between the mesh panels (Fig. 1B). Because the tool tapered it was possible to insert the thinner end into the small gap and push the tool until it became wedged by its increasing thickness. This was a much quicker and easier process than balancing the tool inside the cage lattice and it also secured the tool more effectively. Riau secured the tool in the narrower gap in the following and remaining enrichment trials (~5).

It is unlikely that securing the tool was learnt from previous tool-use enrichment activities that Riau often received. First, Riau had never been observed securing tools during these activities, or on any other occasion. Second, he had never received any previous tool-use enrichment activities in which the rewards were temporarily inaccessible leading to waiting periods. Third, Riau did not hang up the large tool from the beginning of the enrichment session. This would have been expected if he had already learnt to secure tools in this way. Instead, securing the tool occurred spontaneously in the middle of the enrichment session and after he had repeatedly held the tool or dropped it on the floor during the waiting periods. Moreover, when Riau first started to secure the tool, the initial method of balancing the tool inside the cage lattice was cruder than the more efficient method he developed later of wedging the tool into a much narrower gap. Not only was this an easier and quicker way of securing the tool, but it secured it much more robustly than the initial cruder method. And Riau never reverted to securing the tool inside the cage lattice once he had developed the more robust method. This factor, along with the development of securing the tool within even narrower gaps, is therefore a good contender for insightful problem solving^14–16^.

#### Experiment 1

Experiment 1 was conducted three days after the enrichment task. The enrichment set-up was used but the procedure differed in the following ways: in full view of Riau, 12 pieces of rewards (apple and orange) were placed inside a lidless opaque container. On the first trial, and whilst Riau was watching, the experimenter placed two of the rewards inside the ring and gave Riau the large tool. This was the same tool that Riau had made to rake-in the ring in the enrichment session and it was used in all the “Large Tool” trials. The experimenter then stood 4 m from the table and faced Riau. Once Riau had raked-in the ring and pulled both rewards across the mesh with his fingers, the experimenter turned his back to Riau and held the container to his chest which occluded it from Riau’s view. After 1 min, the experimenter turned to face Riau and followed the same procedure as in trial 1 (bar giving Riau the tool as he already had it in his possession). When the last rewards were placed in the ring (trial 6) the experimenter showed Riau the inside of the empty container and inverted it to make it clear there were no rewards left inside. The same procedure was then used as in the previous trials. Riau received 12 sessions, each consisting of 6 trials. One session was conducted per day for 12 consecutive days.

#### Experiment 2

In experiment 2, the same set-up and procedure as in experiment 1 was used with the following two exceptions: (1) in addition to the large tool sessions, Riau was given test sessions in which he was only presented with a small and lighter tool that could also be used to rake in the rewards, and (2) the rewards were placed out of Riau’s reach directly on the table instead of inside the metal ring. This was because the small tool was not strong enough to be used to move the heavy ring. Riau received two conditions: (1) Large Tool, and (2) Small Tool. On the first trial of the Large Tool condition, Riau was given the same large tool that was used in experiment 1 whereas on the first trial of the Small Tool condition he was given a smaller tool (weighing 15 g, 35 cm long and 0.5 cm in diameter). This tool was manufactured by the experimenter from the type of branches that Riau receives daily as a diet supplement and for making tools to rake out rewards from enrichment devices. Prior to the experiment, and out of Riau’s sight, the experimenter had checked that the small tool could easily be inserted and secured in the narrow gap of the cage wall.

Riau received 6 sessions of the Large Tool condition in which he could only use the large tool to rake in the rewards. And he received 6 sessions of the Small Tool condition in which he could only use the small tool to rake in the rewards. Each session consisted of 6 trials. The two conditions were alternated over 12 consecutive days.

### Data scoring and analyses

#### Quantitative Analysis

The experimenter video-recorded all experimental trials. Each video trial was coded to establish if the subject secured the tool within the 1-minute waiting period. Securing the tool was defined as inserting the tool into the narrow gap and then releasing it so that the tool was left hanging. A second observer independently scored 60% of the video trials. Inter-observer reliability was excellent with 100% agreement between the experimenter and second coder (Cohen’s k = 1.0, N = 43).

In experiment 1, I used an exact test to assess the differences between securing the tool when it could be predicted that the tool would be required to rake-in forthcoming rewards (Hang-up Tool trials) or not required as no rewards were forthcoming (Discard Tool trials). Each test session consisted of five replicates of the Hang-up Tool trial (trials 1-5) and one trial of the Discard Tool trial (6^th^ and last trial). I used an exact chi-square test to compare rates of tool securing between the overall number of Hang-up Tool trials and the overall number of Discard Tool trials. The Chi-square analysis assumes independence of observations within a session, which cannot be guaranteed because of potential effects, such as learning. In a second analysis I therefore compared the responses of the test result of the first Hang-up Tool trial only with the score of the Discard Tool (trial 6) of the same session using an exact McNemar test. This test accounts for the matched design and controls for any potential temporal order effects.

Experiment 2 was analysed in the same way as experiment 1. Inter-observer reliability was excellent with 100% agreement between the experimenter and second coder (Cohen’s k = 1.0, N = 43).

#### Qualitative Analysis

Each video trial of experiment 1 and 2 was analysed qualitatively to assess: (1) LEAVE/STAY behaviour (2) WAITING behaviour, AND (3) TOOL HOLD/DROP behaviour. LEAVE/STAY behaviour was assessed by observing if Riau stayed at the test table or left during the 1-minute waiting period that commenced after he had raked-in the rewards of each trial. When Riau stayed at the test table, his waiting behaviour was assessed during the 1-minute waiting period to check if Riau faced the experimenter and whether Riau made any food-begging gestures towards the experimenter, such as holding out his hand. TOOL HOLD/DROP behaviour was assessed when Riau did not hang up the tool and was classed as Riau either holding onto the tool or dropping it during the 1-minute period after he had raked in the rewards.

A second observer independently scored 60% of the video trials. Inter-observer reliability was excellent with 100% agreement between the experimenter and the second coder: LEAVE/STAY behaviour (Cohen’s k = 1.0, N = 86), WAITING behaviour (Cohen’s k = 1.0, N = 86), and TOOL HOLD/DROP behaviour (Cohen’s k = 1.0, N = 86).

All data generated and analysed during the current study are available from the author.

## Electronic supplementary material

Riau’s trial-by-trial behaviour

video sample of experiment
