# Supplementary material for: An Orangutan Hangs Up a Tool for Future Use
Source: Sci Rep. 2018 Aug 27;8:12900. doi: 10.1038/s41598-018-31331-7 (PMC6110832; doi:10.1038/s41598-018-31331-7)
Supplement: Supplementary file 1 — Riau’s trial-by-trial behaviour [file 41598_2018_31331_MOESM1_ESM.docx]

**An Orangutan Hangs Up a Tool for Future Use**

Author: Nicholas J. Mulcahy

**Supporting Information.**

1) Video sample of Riau hanging up the large tool in trial 5 and discarding the same tool in the final trial.

2) Riau’s trial-by-trial behaviour (begging/no begging and leave/stay) and tool-use actions (hang up/hold/discard tool) during the 1-minute period after he had raked-in the food rewards in experiment 1 and 2.

| Experiment 1  Riau was only provided with the Large Tool in all 12 sessions | | | |
| --- | --- | --- | --- |
| Session 1 | Did Riau make any food-begging gestures? | Did Riau stay at the testing table looking in the general direction of the experimenter, or did he leave the testing area? | Did Riau hold on to the tool, hang the tool up, or discard tool? |
| 1 | No | Stayed | Hung tool |
| 2 | No | Stayed | Hung tool |
| 3 | No | Stayed | Hung tool |
| 4 | No | Stayed | Hung tool |
| 5 | No | Stayed | Hung tool |
| 6 | No | Left | Discarded tool |
| Session 2 |  |  |  |
| 1 | No | Stayed | Hung tool |
| 2 | No | Stayed | Hung tool |
| 3 | No | Stayed | Hung tool |
| 4 | No | Stayed | Hung tool |
| 5 | No | Stayed | Hung tool |
| 6 | No | Left | Discarded tool |
| Session 3 |  |  |  |
| 1 | No | Stayed | Hung tool |
| 2 | No | Stayed | Hung tool |
| 3 | No | Stayed | Hung tool |
| 4 | No | Stayed | Hung tool |
| 5 | No | Stayed | Hung tool |
| 6 | No | Left | Discarded tool |
| Session 4 |  |  |  |
| 1 | No | Stayed | Hung tool |
| 2 | No | Stayed | Hung tool |
| 3 | No | Stayed | Hung tool |
| 4 | No | Stayed | Hung tool |
| 5 | No | Stayed | Hung tool |
| 6 | No | Left | Discarded tool |
| Session 5 |  |  |  |
| 1 | No | Stayed | Hung tool |
| 2 | No | Stayed | Hung tool |
| 3 | No | Stayed | Hung tool |
| 4 | No | Stayed | Hung tool |
| 5 | No | Stayed | Hung tool |
| 6 | No | Left | Discarded tool |
| Session 6 |  |  |  |
| 1 | No | Stayed | Held tool |
| 2 | No | Stayed | Hung tool |
| 3 | No | Stayed | Hung tool |
| 4 | No | Stayed | Hung tool |
| 5 | No | Stayed | Hung tool |
| 6 | No | Left | Discarded tool |
| Session 7 |  |  |  |
| 1 | No | Stayed | Hung tool |
| 2 | No | Stayed | Hung tool |
| 3 | No | Stayed | Hung tool |
| 4 | No | Stayed | Hung tool |
| 5 | No | Stayed | Hung tool |
| 6 | No | Left | Discarded tool |
| Session 8 |  |  |  |
| 1 | No | Stayed | Hung tool |
| 2 | No | Stayed | Hung tool |
| 3 | No | Stayed | Hung tool |
| 4 | No | Stayed | Hung tool |
| 5 | No | Stayed | Hung tool |
| 6 | No | Left | Discarded tool |
| Session 9 |  |  |  |
| 1 | No | Stayed | Hung tool |
| 2 | No | Stayed | Hung tool |
| 3 | No | Stayed | Hung tool |
| 4 | No | Stayed | Hung tool |
| 5 | No | Stayed | Hung tool |
| 6 | No | Left | Discarded tool |
| Session 10 |  |  |  |
| 1 | No | Stayed | Hung tool |
| 2 | No | Stayed | Hung tool |
| 3 | No | Stayed | Hung tool |
| 4 | No | Stayed | Hung tool |
| 5 | No | Stayed | Hung tool |
| 6 | No | Left | Discarded tool |
| Session 11 |  |  |  |
| 1 | No | Stayed | Hung tool |
| 2 | No | Stayed | Hung tool |
| 3 | No | Stayed | Hung tool |
| 4 | No | Stayed | Hung tool |
| 5 | No | Stayed | Hung tool |
| 6 | No | Left | Discarded tool |
| Session 12 |  |  |  |
| 1 | No | Stayed | Hung tool |
| 2 | No | Stayed | Hung tool |
| 3 | No | Stayed | Hung tool |
| 4 | No | Stayed | Hung tool |
| 5 | No | Stayed | Hung tool |
| 6 | No | Left | Discarded tool |
| Experiment 2  Riau tested with 6 sessions in which he was only provided with the Small Tool and 6 sessions in which he was only provided with the Large Tool | | | |
| Session 1  SMALL TOOL | Did Riau make any food-begging gestures? | Did Riau stay at the testing table looking in the general direction of the experimenter, or did he leave the testing area? | Did Riau hold on to the tool, hang the tool up, or discard tool? |
| 1 | No | Stayed | Held tool |
| 2 | No | Stayed | Held tool |
| 3 | No | Stayed | Held tool |
| 4 | No | Stayed | Held tool |
| 5 | No | Stayed | Held tool |
| 6 | No | Left | Held tool (left carrying tool) |
| Session 2 LARGE TOOL |  |  |  |
| 1 | No | Stayed | Hung tool |
| 2 | No | Stayed | Hung tool |
| 3 | No | Stayed | Hung tool |
| 4 | No | Stayed | Hung tool |
| 5 | No | Stayed | Hung tool |
| 6 | No | Left | Discarded tool |
| Session 3 SMALL TOOL |  |  |  |
| 1 | No | Stayed | Held tool |
| 2 | No | Stayed | Held tool |
| 3 | No | Stayed | Held tool |
| 4 | No | Stayed | Held tool |
| 5 | No | Stayed | Held tool |
| 6 | No | Stayed | Held tool |
| Session 4 LARGE TOOL |  |  |  |
| 1 | No | Stayed | Hung tool |
| 2 | No | Stayed | Hung tool |
| 3 | No | Stayed | Hung tool |
| 4 | No | Stayed | Hung tool |
| 5 | No | Stayed | Hung tool |
| 6 | No | Left | Discarded tool |
| Session 5 SMALL TOOL |  |  |  |
| 1 | No | Stayed | Held tool |
| 2 | No | Stayed | Held tool |
| 3 | No | Stayed | Held tool |
| 4 | No | Stayed | Held tool |
| 5 | No | Stayed | Held tool |
| 6 | No | Stayed | Held tool |
| Session 6 LARGE TOOL |  |  |  |
| 1 | No | Stayed | Hung tool |
| 2 | No | Stayed | Hung tool |
| 3 | No | Stayed | Hung tool |
| 4 | No | Stayed | Hung tool |
| 5 | No | Stayed | Hung tool |
| 6 | No | Left | Discarded tool |
| Session 7 SMALL TOOL |  |  |  |
| 1 | No | Stayed | Held tool |
| 2 | No | Stayed | Held tool |
| 3 | No | Stayed | Held tool |
| 4 | No | Stayed | Held tool |
| 5 | No | Stayed | Held tool |
| 6 | No | Left | Held tool (left carrying tool) |
| Session 8 LARGE TOOL |  |  |  |
| 1 | No | Stayed | Hung tool |
| 2 | No | Stayed | Hung tool |
| 3 | No | Stayed | Hung tool |
| 4 | No | Stayed | Hung tool |
| 5 | No | Stayed | Hung tool |
| 6 | No | Left | Discarded tool |
| Session 9 SMALL TOOL |  |  |  |
| 1 | No | Stayed | Held tool |
| 2 | No | Stayed | Held tool |
| 3 | No | Stayed | Held tool |
| 4 | No | Stayed | Held tool |
| 5 | No | Stayed | Held tool |
| 6 | No | Left | Held tool (left carrying tool) |
| Session 10 LARGE TOOL |  |  |  |
| 1 | No | Stayed | Hung tool |
| 2 | No | Stayed | Hung tool |
| 3 | No | Stayed | Hung tool |
| 4 | No | Stayed | Hung tool |
| 5 | No | Stayed | Hung tool |
| 6 | No | Left | Hung tool |
| Session 11 SMALL TOOL |  |  |  |
| 1 | No | Stayed | Held tool |
| 2 | No | Stayed | Held tool |
| 3 | No | Stayed | Held tool |
| 4 | No | Stayed | Held tool |
| 5 | No | Stayed | Held tool |
| 6 | No | Left | Held tool (left carrying tool) |
| Session 12 LARGE TOOL |  |  |  |
| 1 | No | Stayed | Hung tool |
| 2 | No | Stayed | Hung tool |
| 3 | No | Stayed | Hung tool |
| 4 | No | Stayed | Hung tool |
| 5 | No | Stayed | Hung tool |
| 6 | No | Left | Discarded tool |
